# Uncovering a Genetic Diagnosis in a Pediatric Patient by Whole Exome Sequencing: A Modeling Investigation in Wiedemann–Steiner Syndrome

**DOI:** 10.3390/genes15091155

**Published:** 2024-09-01

**Authors:** Ighli di Bari, Caterina Ceccarini, Maria Curcetti, Carla Cesarano, Anna-Irma Croce, Iolanda Adipietro, Maria Grazia Gallicchio, Grazia Pia Palladino, Maria Pia Patrizio, Benedetta Frisoli, Rosa Santacroce, Maria D’Apolito, Giovanna D’Andrea, Ombretta Michela Castriota, Ciro Leonardo Pierri, Maurizio Margaglione

**Affiliations:** 1 Medical Genetics, Department of Clinical and Experimental Medicine, University of Foggia, 71122 Foggia, Italy; ighli.dibari@unifg.it (I.d.B.); cceccarini@ospedaliriunitifoggia.it (C.C.); maria.curcetti@unifg.it (M.C.); ccesarano@ospedaliriunitifoggia.it (C.C.); aicroce@ospedaliriunitifoggia.it (A.-I.C.); iadipietro@ospedaliriunitifoggia.it (I.A.); g.mgrazia969@gmail.com (M.G.G.); gpalladino@ospedaliriunitifoggia.it (G.P.P.); mpatrizio@ospedaliriunitifoggia.it (M.P.P.); benedetta_frisoli.603094@unifg.it (B.F.); rosa.santacroce@unifg.it (R.S.); maria.dapolito@unifg.it (M.D.); giovanna.dandrea@unifg.it (G.D.); 2 Neuropsychiatry for Child and Adolescent Unit, Department of Woman and Child, Policlinico Riuniti, 71122 Foggia, Italy; ombrettacastriota@gmail.com; 3 Department of Pharmacy-Pharmaceutical Sciences, University of Bari “Aldo Moro”, 70125 Bari, Italy; ciro.pierri@uniba.it

**Keywords:** whole exome sequencing, modeling, Wiedemann–Steiner syndrome, *KMT2A* gene

## Abstract

**Background:** Wiedemann–Steiner syndrome (WSS), a rare autosomal-dominant disorder caused by haploinsufficiency of the *KMT2A* gene product, is part of a group of disorders called chromatinopathies. Chromatinopathies are neurodevelopmental disorders caused by mutations affecting the proteins responsible for chromatin remodeling and transcriptional regulation. The resulting gene expression dysregulation mediates the onset of a series of clinical features such as developmental delay, intellectual disability, facial dysmorphism, and behavioral disorders. **Aim of the Study:** The aim of this study was to investigate a 10-year-old girl who presented with clinical features suggestive of WSS. **Methods:** Clinical and genetic investigations were performed. Whole exome sequencing (WES) was used for genetic testing, performed using Illumina technology. The bidirectional capillary Sanger resequencing technique was used in accordance with standard methodology to validate a mutation discovered by WES in all family members who were available. Utilizing computational protein modeling for structural and functional studies as well as in silico pathogenicity prediction models, the effect of the mutation was examined. **Results:** WES identified a de novo heterozygous missense variant in the *KMT2A* gene KMT2A(NM_001197104.2): c.3451C>G, p.(Arg1151Gly), absent in the gnomAD database. The variant was classified as Likely Pathogenetic (LP) according to the ACMG criteria and was predicted to affect the CXXC-type zinc finger domain functionality of the protein. Modeling of the resulting protein structure suggested that this variant changes the protein flexibility due to a variation in the Gibbs free energy and in the vibrational entropy energy difference between the wild-type and mutated domain, resulting in an alteration of the DNA binding affinity. **Conclusions:** A novel and de novo mutation discovered by the NGS approach, enhancing the mutation spectrum in the *KMT2A* gene, was characterized and associated with WSS. This novel *KMT2A* gene variant is suggested to modify the CXXC-type zinc finger domain functionality by affecting protein flexibility and DNA binding.

## 1. Introduction

Wiedemann–Steiner syndrome (WSS) is a rare autosomal dominant disorder with a prevalence of <1 in 1,000,000 caused by harmful heterozygous variants of the *KMT2A* gene (*159555) located on chromosome 11q23.3. The *KMT2A* gene, also known as the *MLL* gene, consists of 36 exons spanning 16.6 Kb and encodes a histone lysine methyltransferase composed of 3969 amino acids. The *KMT2A* protein includes several functional domains: three DNA binding AT hooks, a CXXC zinc finger domain, four PHD zinc finger domains, a BD domain, a FYRN domain, a TAD motif, a FYRC domain, a WIN motif, and a SET domain [1]. As of now, 491 different *KMT2A* variants have been documented in the Human Gene Mutation Database (https://www.hgmd.cf.ac.uk/ac/gene.php?gene=KMT2A) (accessed on 30 May 2024) as causing WSS [2]. KMTs transfer methyl groups from S-adenosylmethionine to lysine residues on histone tails, with a particular focus on the histone H3 tail. Unlike acetyltransferases (HATs), which have broader activity, KMTs are more specific and typically modify only one or two lysines on a single histone [3]. Lysine-specific methyltransferase 2A (*KMT2A*) methylates histone H3 at lysine 4 (H3K4me) and plays a role in chromatin remodeling, serving as a “writer” in the epigenetic machinery [4]. WSS patients exhibit complicated and diverse symptoms due to the *KMT2A* gene, which regulates well-characterized targets such as *HOX* genes and *WNT* gene expression and plays a crucial role in early development and hematopoiesis. Common characteristics of WSS include developmental delay, intellectual disability, short stature, facial dysmorphism, and hairy elbows. Patients with WSS can also present with feeding difficulties, epilepsy, ocular abnormalities, congenital heart disease, musculoskeletal problems, genitourinary anomalies, endocrinologic issues, immunologic dysfunction, and behavioral disorders [5,6,7]. Strong evidence supporting the autosomal-dominant inheritance pattern of WSS is provided by the involvement of haploinsufficiency of the *KMT2A* gene in its pathogenesis. As a result, genetic testing is used to confirm the diagnosis of WSS after it has been established based on typical clinical symptoms [8].

A 10-year-old child with a novel and de novo *KMT2A* mutation and a diagnosis of WSS is the subject of this investigation. After in silico and pedigree analyses were performed to assess the variant’s potential impact on pathology, the variant was classified as “Likely Pathogenic”. Investigating the potential consequences of the mutation on protein functionality using a modeling approach can provide valuable insights into the mechanisms of pathogenicity and it can be useful in evaluating other people who exhibit symptoms of chromatinopathy.

## 2. Materials and Methods

### 2.1. Clinical and Genetic Investigation

A 10-year-old girl who experienced an epileptic episode was referred to the Neuropsychiatric Unit for Children and Adolescents at Policlinico Riuniti Hospital in Foggia for clinical and genetic evaluations, including abdominal ultrasonography, cardiac examination, waking and sleep electroencephalography (EEG), physical and neurological assessments, and brain magnetic resonance imaging (MRI). The cognitive disability was assessed with the WISC-IV test (Wechsler Intelligence Scale for Children 4° edition). All available family members underwent detailed medical examinations. The clinical and genetic studies complied with the Helsinki declaration. Genetic counselling was provided, and informed consent for testing was obtained from all participants. The data presented in this manuscript were anonymized, and the study received approval from the local Ethics Committee (Protocol code 17/CE/2014).

### 2.2. FMR1 Analysis

A capillary electrophoresis technique was used to detect FMR1 expanded alleles (FraxA 1 Kit, Experteamsrl, Venice, Italy).

### 2.3. Karyotype

Karyotype analysis was carried out on QFQ-banded phytohemagglutinin-stimulated lymphocyte metaphases following standard protocols suggested by the manufacturers.

### 2.4. SNP-Array 850K

SNP-array analysis was performed using the Illumina Infinium CytoSNP-850K v1.2 microarray, scanned with an Agilent G5761A microarray scanner (Agilent Technologies, Inc., Santa Clara, CA, USA) and analyzed by BlueFuse Multi v4.5 (Illumina, Inc., San Diego, CA, USA), according to the standard protocols of the manufacturer.

### 2.5. Exome Sequencing

According to our internal procedures, in the absence of a positive family history, whole exome sequencing (WES) is only performed on the proband. Standard protocols were used to obtain genomic DNA from the proband’s whole blood. The Illumina DNA Prep with Enrichment protocol (San Diego, CA, USA) was utilized to produce the sample libraries by isolating the genomic DNA from peripheral blood. Using an Illumina NextSeq 550^®^ Sequencing System (San Diego, CA, USA), all coding regions and exon–intron junctions were sequenced (±50 bps). At an average depth of 99×, over 30 times were covered in about 99% of the targeted locations. Clean sequencing reads from the NextSeq 550 System were used to analyze the data and the Burrows–Wheeler Aligner (v.0.7.12) was used to match the results to the human reference genome, GRCh37/hg19. The Genome Analysis Toolkit (GATK 1.6) was utilized for variant calling and the BaseSpace Variant Interpreter Annotation Engine 3.15.0.0 (Illumina, San Diego, CA, USA) was utilized for annotation and analysis. Variants were annotated according to the Human Genome Variation Society (HGVS) guidelines and classified according to the criteria of the American College of Medical Genetics and Genomics (ACMG). The pathogenicity of all rare genetic variants was classified following the ACMG 2015 guidelines. Further investigations were conducted to pinpoint variants that are pathogenic, likely pathogenic, or of uncertain significance (VUS) associated with the phenotype.

#### Genetic Testing and Prioritization of Variants

We implemented the following filtration steps and various strategies were employed to reduce the quantity of potentially harmful genetic artifacts: filtering based on a quality score of >Q30 and PASS filter; removing variants with a minor allele frequency of >0.01; removing variants outside of the coding regions or variants that are synonymous with coding variants; filtering the data for novelty by comparison with published studies, including the Single Nucleotide Polymorphism Database (http://ncbi.nlm.nih.gov/snp (accessed on 30 May 2024)), the Genome Aggregation Database (http://gnomad.broadinstitute.org/ (accessed on 30 May 2024)), the 1000 Genomes Project (https://www.internationalgenome.org/1000-genomes-browsers/index.html (accessed on 30 May 2024)), and other published studies; choosing variants that segregate in accordance with the assumed pattern of inheritance; and querying disease databases, such as ClinVar (https://ncbi.nlm.nih.gov/clinvar/ (accessed on 30 May 2024)), OMIM (http://www.omim.org (consulted on 30 May 2024)), and the Human Gene Mutation Database locus-specific database (http://www.hgvs.org/ (accessed on 30 May 2024)) to refine the prioritization of candidate gene variants. Based on the linked phenotype described in the literature and the function of the corresponding protein, several of them were eliminated. Following this stage of functional annotation, 301 variants were selected for further investigation.

### 2.6. Sanger Sequencing

Following the recommendations of the American College of Medical Genetics and Genomics, Sanger sequencing was conducted on a SeqStudio™ Genetic Analyzer System from Applied Biosystems™ (Waltham, MA, USA) on amplicons of the exon of interest generated with custom primers by Primer3 v4.1.0 (For: tgaattcagtactcccttgga, Rev: tctctgcaacaaactaggga) to validate the WES results. NCBI GenBank accession number NM_001197104 and 2 UniProt identifier Q03164-3 were the reference sequences. Exon 5 of the *KMT2A* gene (NM_001197104.2) was amplified using specific custom primers and the PCR products were sequenced with BigDye Terminator v.3.1 (Thermo Fisher Scientific, Waltham, MA, USA) according to standard protocols. The prioritized mutation was validated in probands and then investigated in other family members when DNA was available for segregation analysis.

### 2.7. In Silico Analysis and Protein Structure Modeling

To further prioritize candidate gene variants, nonsynonymous amino acid substitutions and splicing variants were selected based on their annotations related to protein functionality and phenotype information. Various bioinformatic tools were employed to assess the pathogenicity of these genetic variants and their impact on protein function. The impact of the p.Arg1151Gly substitution was investigated using in silico pathogenicity prediction techniques AlphaMissense [9], Varity [10], Mutation Taster [11], DANN [12], MetaLR [13], Revel [14], PolyPhen-2 [15], SIFT [16], and CADD [17]. Employing a wide array of these tools is essential for obtaining a robust and reliable assessment of the variant’s potential pathogenicity. Each tool utilizes different algorithms and databases, offering unique insights and increasing the overall confidence in the prediction outcomes. By integrating the results from multiple tools, we can better account for various aspects of the potential impact of the gene variation, such as structural alterations, evolutionary conservation, and functional disruptions. The effect of the variant was evaluated using in silico prediction and protein modeling by using computational techniques DynaMut2, mCSM-DNA, PremPDI, Swiss-Pdb Viewer, and PyMOL v3.0.3 (https://www.pymol.org/ (accessed on 30 May 2024)), that predict changes in protein stability with the input files suggested by PSIPRED-pDomTREADER and ProtVar v2.1 [18,19,20,21,22,23]. PSI-blast-based secondary structure PREDiction (PSIPRED), is a protein structure investigation technique based on the use of an artificial neural network machine learning method and on the fold recognition algorithm pDomTHREADER, which is part of the latest implementation of the GenTHREADER, an efficient and reliable protein fold recognition method for genomic sequences, accessible via the PSIPRED web server. These tools are crucial for predicting changes in protein stability and interactions, offering a detailed understanding of how the p.Arg1151Gly substitution might alter the structure and function of the protein ensuring accurate and consistent data across the different modeling techniques, enhancing the reliability and depth of findings.

## 3. Results

### 3.1. Proband Clinical Characteristics

**Figure 1:**
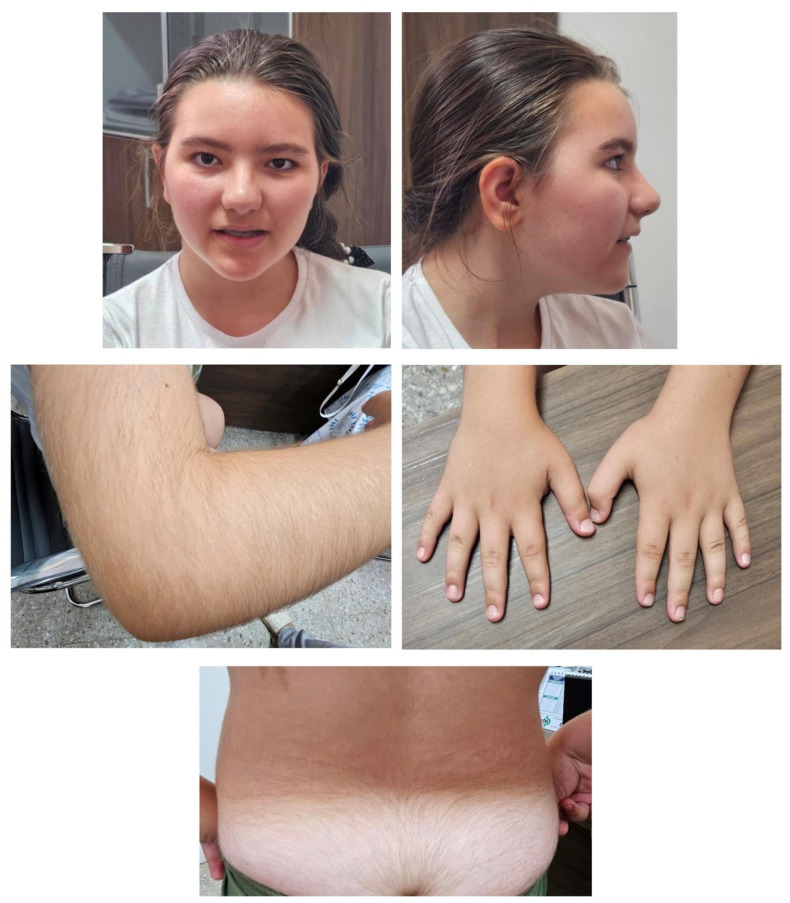
Patient’s characteristics. Features include mild facial dysmorphisms (hypertelorism, epicanthus, wide nasal bridge, and low-set ears), stubby and puffy hands, and hypertrichosis.

A 10-year-old proband was referred to the Policlinico Riuniti Hospital of Foggia Neuropsychiatric Unit for Children and Adolescents because of a first epileptic episode. The patient appeared in good general condition and apyretic. At the neurological physical examination, she showed height according to age, mild facial dysmorphisms (hypertelorism, epicanthus, wide nasal bridge, and low-set ears), myopia, dental anomalies (malocclusion and needing a palate expander), hypertrichosis, stubby and puffy hands, skeletal anomalies (prominent cervical vertebrae), motor impairment, low pain threshold, and minimal motor and speech disorders (Figure 1). The patient was the first daughter of two children, born after a normal pregnancy at the 39th week of gestation, through a normal spontaneous vaginal delivery. The stages of psycho-evolutionary development were reached according to age, with reported memory difficulties and sufficient academic performance. A positive family history for cerebral aneurysm (maternal aunt) was reported. Subsequent outpatient checks confirmed the presence of further episodes characterized by staring and the loss of contact for a few minutes, which resolved spontaneously with epileptiform anomalies. The patient attended the first class of a lower secondary school with scholastic support (18 h/week), with an individualized educational plan for minimum objectives, and she was assuming antiepileptic therapy. She was subjected to instrumental investigations: brain MRI with evidence of a slightly low position of the right cerebellar tonsil and wake and sleep electroencephalography (EEG), which highlighted bihemispheric epileptiform anomalies clearly increased by light sleep. Blood chemistry tests, electrocardiogram, and abdominal ultrasound showed normal findings. The neuropsychological evaluation confirmed the intellectual disability of moderate degree on the WISC-IV test. While FMR1, Karyotype, and SNP-array 850K analyses were normal, whole exome sequencing (WES) revealed a de novo variant (c.3451C>G) in heterozygosity in the *KTM2A* gene, likely responsible for the patient’s phenotype, evocative of Wiedemann–Steiner syndrome. Antiepileptic therapy with levetiracetam was therefore started.

### 3.2. Whole-Exome Sequencing Revealed a Mutation Responsible for Wiedemann–Steiner Syndrome

**Figure 2:**
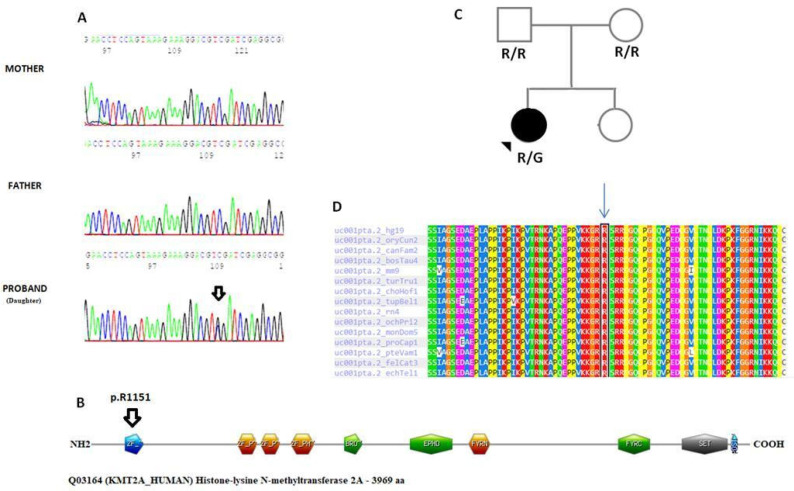
(**A**) Electropherograms showing the c.3451C>G variant in the index case. The arrow shows the nucleotide substitution. (**B**) Schematic representation of the Histone lysine N-methyltransferase 2A (UniProt Q03164) showing the localization of the variant p.R1151G (highlighted with the arrow) found in the proband. The various domains are shown in sequential order from NH2 to COOH: CXXC-type zinc finger (CxxC), plant homeodomain (PHD), Bromodomain, extended plant homeodomain (ePHD), FY-rich N-terminal (FYRN), FY-rich C-terminal (FYRC), Su(Var)3-9 enhancer-of-zestetrithorax (SET) and post-SET. (**C**) Pedigree of proband with mutation in *KMT2A* and her family. Circles show females, squares show males. An arrowhead indicates the proband. (**D**) Alignment of the CXXC-type zinc finger domain (the amino acid location p.1151 is showed in red and indicated by a blue arrow) along different species boxed in light blue.

Assuming a dominant mode of inheritance, WES of the index case revealed a heterozygous mutation in *KMT2A* gene exon 5. The variant found was a nucleotide substitution (c.3451C>G), which predicted a missense amino acid alteration where a glycine would take the place of an arginine (p.R1151G). The ACMG (2015) guidelines classify this variation as Likely Pathogenic (LP)—Class IV, with the following criteria: PM1, PM5, PP3, PM2 [24]. The variant identified was previously unreported and not documented in GnomAD. A number of bioinformatics prediction tools provide ratings based on calculations made to assess the pathogenicity of the detected variant. The results of in silico studies suggested that the variation might have harmful effects (Supplementary Table S1). The p.R1151G variant, which was discovered by WES, was confirmed in the proband, and examined in the parents using direct capillary Sanger sequencing in accordance with normal methodology. Following the validation of Sanger sequencing and genotyping of all family members that were available, the *KMT2A* mutation R1151G was found to perfectly align with the phenotype. The variant occurred as a de novo genetic alteration because it was not inherited from her parents. A significant degree of conservation across several species was found when the human protein was compared to ortholog sequences (Figure 2).

**Figure 3:**
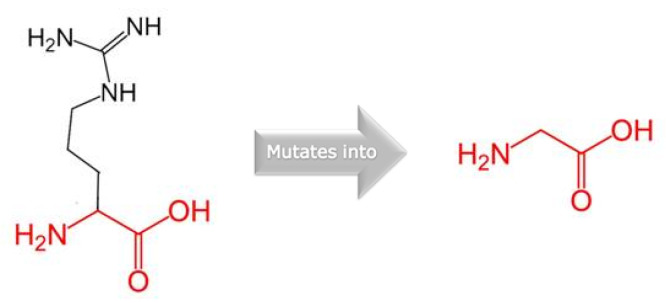
The figure displays the schematic conformation of the original (arginine, R, on the **left**) and the variant (glycine, G, on the **right**) amino acid. The backbone, which is the same for each amino acid, is colored in red. The side chain, unique for each amino acid, is colored in black.

The functional CXXC-type zinc finger domain (InterPro: IPR002857) contains the amino acid residue p.1151. Additionally, we examined the structural data using the web server HOPE. The substitution of an arginine (R) with a glycine (G) resulted in chemical and physiological changes. The G variant residue is apolar (neutral) and smaller than the wild-type R residue, which is positively charged and with a large side chain. Furthermore, the G variant amino acid confers flexibility to local secondary structures and the lack of the positively charged sidechain of R results in a loss of hydrogen bonds, which disturbs the native folding [25] (Figure 3).

**Figure 4:**
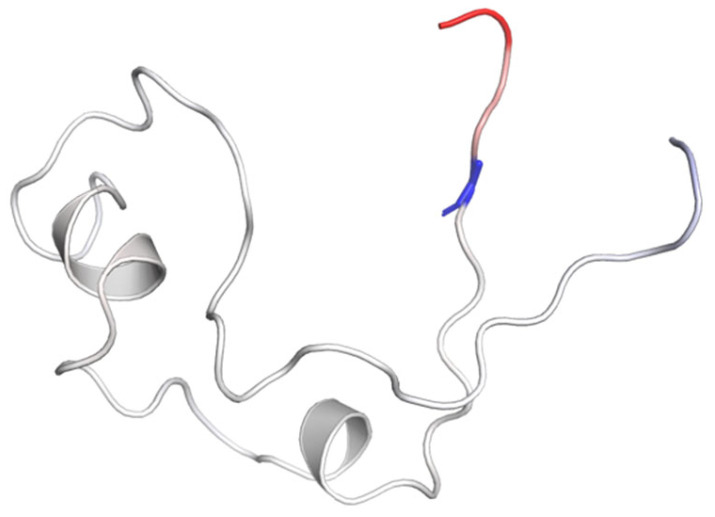
Amino acids are color-coded based on the change in vibrational entropy after mutation, with blue indicating increased rigidity and red indicating enhanced flexibility of the structure.

The PSIPRED-pDomTHREADER fold recognition tool was employed to search for KMT2A structures to investigate the putative pathogenic effect of the p.R1151G variant. Results of the PSIPRED-pDomTHREADER tool highlighted the NMR structure of *KMT2A* (residues p.1147–1203), with the PDB code 2JYI. We used the DynaMut2 (https://biosig.lab.uq.edu.au/dynamut2/ (accessed in 30 May 2024)) web server to assess the impact of the discovered mutation on the residual flexibility. This tool predicted a vibrational entropy energy change between the wild-type (R1151) and the variant (G1151) proteins, resulting in a change in the flexibility due to variations in the Gibbs free energy (ΔΔG: −0.47 kcal/mol) and in the vibrational entropy energy difference (ΔΔSVibENCoM: 0.124 kcal mol^−1^.K^−1^) between the wild-type and the variant domains (Figure 4).

Further investigations by querying ProtVar v2.1, a web resource to investigate single nucleotide variants in humans, identified a crystal structure of the MLL CXXC domain in complex with a CpG DNA (4NW3.pdb) [26]. Consequently, a predictive analysis, performed to assess the impact of the variant on the protein’s binding capacity with nucleic acid using mCSM Protein–DNA, resulted in a destabilizing Predicted Affinity Change (ΔΔG: −1.481 kcal/mol). Furthermore, the consequence of the amino acid substitution on the alteration of binding affinity was also evaluated with PremPDI, a server aimed at mapping pathogenic variations on a structural protein–DNA complex. It calculates the associated changes in binding affinity, determines the deleterious effect of a variation, and produces a mutant structural model. As can be seen from the prediction (Table 1), the variant investigated was causative of the decrease in binding affinity related to the protein–DNA complex.

**Figure 5:**
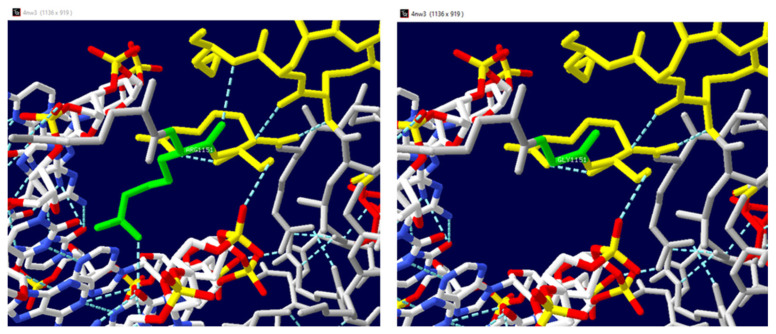
Comparison of the predicted structures of both (**left**) wild-type (R1151) and (**right**) variant (G1151) proteins using the Swiss-Pdb Viewer (https://spdbv.unil.ch/ accessed on 15 May 2024). Hydrogen bonds are displayed in light blue dashed lines in the wild-type and mutant proteins. In the structure, the helices are highlighted in red and the strands in yellow. The R1151 (**left panel**) and G1151 (**right panel**) products are indicated as green sticks. White sticks indicate DNA.

The crystal structure of the MLL CXXC domain in complex with a CpG DNA (4NW3.pdb) was used as a template to investigate the consequences of the p.R1151G variant in amino acid interactions. The Swiss-Pdb Viewer modelling shows the comparison of the structures of both wild-type and predicted mutant proteins (Figure 2) (https://spdbv.unil.ch/ (accessed on 30 May 2024)). The Swiss-Pdb Viewer detected a change in the hydrogen bond interactions between the wild-type amino acid (R1151) and the surrounding amino acids compared to the hydrogen bonds established by the mutated amino acid (G1151) (Figure 5).

**Figure 6:**
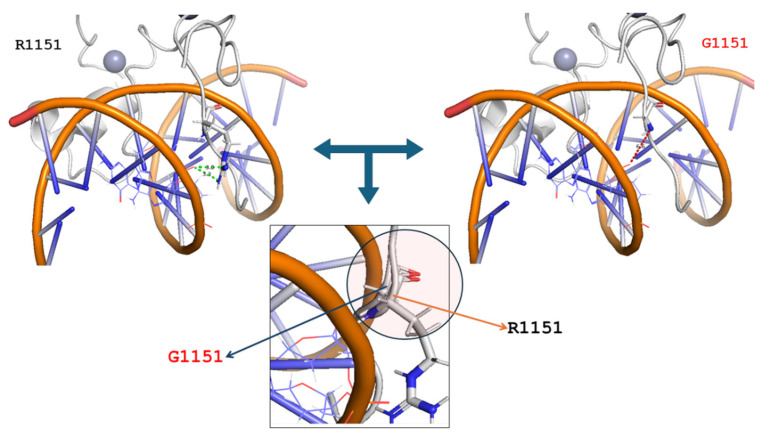
Molecular characterization and 3D modeling of the *KMT2A* mutational profile. KMT2A is indicated by the white illustration, and R1151 or G1151 are depicted as white sticks. CpG DNA is indicated as orange and light pink lines. Zn ions are indicated by grey spheres. In the insert at the bottom of the figure, a slight perturbation of the local secondary structure of the CXXC domain is highlighted by comparing the variant (blue arrow) and wild-type (orange arrow) proteins.

In addition, by using the mutagenesis tool implemented in the PyMOL molecular visualizer (https://www.pymol.org/ (accessed on 30 May 2024)), it was possible to build the structure of the MLL CXXC R1151G variant. The structure of the G1151 variant was compared with the structure of the wild-type MLL CXXC domain (PDB 2KKF). Both the wild-type and variant were reported in complex with the palindromic CpG DNA. The structural comparative analysis allowed us to highlight the increased distance between the reported CpG DNA and the new glycine residue (6.0 Å) as compared to the native arginine (3.3 Å). In addition, it was possible to observe that the introduction of a glycine residue, instead of the native arginine residue, caused a slight perturbation of the local secondary structure of the MLL CXXC domain (Figure 6).

## 4. Discussion

A novel and de novo heterozygous missense variant in the lysine methyltransferase 2A (*KMT2A*) gene was identified. The primary aim of this study was to report this previously undescribed variant and conduct an in silico investigation of its potentially pathogenic consequences. The variant identified, c.3451C>G, was a nucleotide substitution which implicated the substitution of an arginine by a glycine, p.R1151G, altering the hydrogen bond network. Protein folding is largely influenced by hydrogen bonding, particularly when it comes to arranging secondary structures and the protein–water interface [27]. In silico analyses predicted a deleterious effect of the variant on protein structure and functionality. The loss-of-function mutation in the *KMT2A*CxxC domain leads to an increased DNA methylation. The variant occurred in the CXXC domain, which binds unmethylated CpGs, and a loss of capacity of *KMT2A* to bind CpGs is a key factor in WSS [28,29]. *KMT2A* belongs to the large family of proteins called lysine methyltransferases (KMTs) and it comprises 38 exons, including the 5′- and 3′-unstranslated regions (UTR), distributed across a 90,375 bp region at 11q23.3 [30]. The methyl groups on S-adenosylmethionine are transferred to the lysine residues on histone tails, especially the histone H3 tail, by KMTs. The latter are more selective than other epigenetic enzymes like acetyltransferases (HATs) and typically alter one or two lysines on a single histone. The side chain of an amino acid remains electrically charged even when lysines are mono-, bi-, or trimethylated. The methylation states and their placements determine the influence on the chromatin state, or whether it represses or stimulates transcription.

Mutations in genes coding for epigenetic machinery (writers, erasers, and readers) lead to chromatinopathies, causing dominant Mendelian disorders due to altered chromatin and gene expression [31]. These alterations are reversible because they may be undone by erasers to revert their effect on gene expression [32,33,34,35]. Readers have specific expertise for recognizing and interpreting various chemical changes. On lysine 4 of histone 3 (H3K4 me1/2/3), the SET domain possesses methyltransferase activity (mono-, di-, and trimethylation), a post-transcriptional modification (PTM) that oversees epigenetic transcriptional activation [36,37].

The *KMT2A* gene is widely recognized in the literature to be associated with WSS [38]. Also, *KMT2A* has been shown to play a significant role in several functional processes of embryonic development, as demonstrated in animal models, including in zebrafish and mice. For example, a prior zebrafish study has shown that *KMT2A* is necessary for the development of the nervous system in zebrafish embryos. Furthermore, prior studies have shown that total *KMT2A* disruption in mouse embryos results in lethality, and heterozygous animals display a range of symptoms, such as skeletal deformities and growth retardation and memory formation [39,40]. *KMT2A* plays a crucial role in the regulation of intricate behaviors by maintaining appropriate amounts of H3K4me3 at a select few, but crucial, gene promoters linked to emotion and cognition. The differences in clinical phenotypes between homozygous and heterozygous animals clearly point to a pivotal role of dosage-sensitive regulation of the *KMT2A* [41]. Investigations into developmental disorders also included Drosophila melanogaster, in which mutations in the *KMT2A* homolog (trx) resulted in a variety of homeotic changes [42].

### 4.1. Clinical Overlap between WSS and Other Syndromes

The patient investigated shows a de novo missense variant, c.3451C>G, in the *KMT2A* gene never described before, with typical features of WSS such as facial dysmorphisms, moderate intellectual disability, and hypertrichosis, and less frequent features such as epilepsy, stubby hands, myopia, motor impairment, and dental and skeletal anomalies [43]. To date, low pain threshold has never been reported in any WSS patient (nor in any other chromatinopathy) and may not be associated with the syndrome. WSS shares certain clinical characteristics with other syndromes. A differential diagnosis based solely on the clinical phenotype is challenging because WSS has a broad range of phenotypic traits, according to the idea that germline variations impacting the epigenetic machinery provide molecular consequences (chromatin state modifications), which can be found in a series of clinical disorders. In particular, the main WSS phenotypes overlapping with other chromatinopathies (e.g., Coffin–Siris syndrome, Kabuki syndrome, Cornelia De Lange syndrome, Rubinstein–Taybi syndrome) are growth deficiency, neurological/cognitive impairment, and similar dysmorphisms and limb anomalies for Rubinstein–Taybi syndrome; similar facial features and/or hypertrichosis for Kabuki syndrome; developmental delay and/or intellectual disability and hypertrichosis for Coffin–Siris syndrome; and thick eyebrows, short stature, moderate to severe developmental delay, and/or intellectual disability and hypertrichosis for Cornelia De Lange syndrome [44,45]. In these syndromes, the causative variants impact a gene involved in regulating epigenetic processes that control the proper balance of chromatin opening and closing at specific sites. As a result, a loss of a key player could perturb the equilibrium leading to similar outcomes or to a spectrum of overlapping clinical conditions. In fact, even at the molecular level, *KMT2A* germinal pathogenic variants are found both in WSS patients and in patients with a clinical diagnosis of several other chromatinopathies [46]. This similarity underscores the complexity of these conditions and highlights the need for careful clinical evaluation to accurately differentiate between them, despite their shared characteristics. Thus, when individuals have traits suggestive of a chromatinopathy, a WES approach plays an important role for the differential analysis and diagnosis [4,47,48]. For instance, identifying a specific missense mutation can provide a clearer genetic diagnosis; this can facilitate early intervention and personalized management strategies tailored to the patient’s specific needs. Additionally, it can contribute to expanding the genotype–phenotype correlations in WSS, thereby improving the overall understanding of the disease and supporting the development of targeted therapies in the future.

### 4.2. Limitations of the Study

The present study has a series of limitations. First, a unique patient is described. The identification of the same gene variant in other patients would strongly support the pathogenicity. In addition, several in silico models were used to evaluate the impact of the new variant on the functionality of the gene product. Experimental validation and functional studies are needed to confirm the predictions and to better understand the effects of the variant identified in the biological context.

## 5. Conclusions

In summary, we reported a de novo missense LP variant c.3451C>G in the *KMT2A* gene that has not been previously described. This variant could modify the binding affinity of protein to DNA. To date, the phenotypic overlap observed among Mendelian disorders involving the epigenetic mechanisms is well known. This likely reflects interconnected pathways involved in the complex regulation of the balance between open and closed chromatin. The findings from the present study can be helpful in assessing other individuals who show features of chromatinopathy and further highlight how crucial it is to adopt a WES approach to obtain a differential diagnosis when a chromatinopathy is suspected.

## Figures and Tables

**Table 1:** Predicted change in binding affinity and mutation classification.

Mutated Chain	Mutation	ΔΔG	Interface	Deleterious
A	R1151G	1.25	Yes	Yes

ΔΔGbind (kcal mol^−1^) is the predicted change in the binding affinity induced by a variant; PremPDI defines a residue to be located on a protein–DNA interface. The PremPDI server classifies a mutation as deleterious if ΔΔGbind is higher or equal to 1.10 kcal mol^−1^.

## Data Availability

The corresponding author may provide the data from this study upon request.

## Supplementary Materials

The following supporting information can be downloaded at: https://www.mdpi.com/article/10.3390/genes15091155/s1, Table S1: In silico predicted pathogenicity of the *KMT2A* variant.
