# Supplementary material for: Uncovering a Genetic Diagnosis in a Pediatric Patient by Whole Exome Sequencing: A Modeling Investigation in Wiedemann–Steiner Syndrome
Source: Genes (Basel). 2024 Sep 1;15(9):1155. doi: 10.3390/genes15091155 (PMC11431573; doi:10.3390/genes15091155)
Supplement: Supplementary file 1 [file genes-15-01155-s001.zip › genes-3154702-supplementary.pdf]

Gene	Nucleotide	AA Change	SIFT	PolyPhen	AlphaMissense	Varity	MT	DANN	MetaLR	CADD	Revel
KMT2S	C/G	R1151G	Deleterious (0)	Probably Damaging (1)	Deleterious (0.996)	Deleterious (0.78)	Deleterious (1)	Deleterious (1)	Deleterious (0.78)	Probably Deleterious (26.2)	Deleterious (0.77)

Supplementary Table S1: In silico predicted pathogenicity of the KMT2A variant.
